# The leaves of the seasoning plant *Litsea cubeba* inhibit the NLRP3 inflammasome and ameliorate dextran sulfate sodium-induced colitis in mice

**DOI:** 10.3389/fnut.2022.871325

**Published:** 2022-07-27

**Authors:** Wei-Ting Wong, Chun-Hsien Wu, Lan-Hui Li, De-Yu Hung, Hsiao-Wen Chiu, Hsien-Ta Hsu, Chen-Lung Ho, Oleg V. Chernikov, Shu-Meng Cheng, Shih-Ping Yang, Chih-Hsin Chung, Kuo-Feng Hua, Chin-Fah Wang

**Affiliations:** ^1^Department of Biotechnology and Animal Science, National Ilan University, Ilan, Taiwan; ^2^Division of Cardiology, Department of Internal Medicine, National Defense Medical Center, Tri-Service General Hospital, Taipei, Taiwan; ^3^Department of Laboratory Medicine, Linsen, Chinese Medicine and Kunming Branch, Taipei City Hospital, Taipei, Taiwan; ^4^Department of Pathology, National Defense Medical Center, Tri-Service General Hospital, Taipei, Taiwan; ^5^Division of Neurosurgery, Taipei Tzu Chi Hospital, Buddhist Tzu Chi Medical Foundation, New Taipei City, Taiwan; ^6^School of Medicine, Buddhist Tzu Chi University, Hualien, Taiwan; ^7^Division of Wood Cellulose, Taiwan Forestry Research Institute, Taipei, Taiwan; ^8^G.B. Elyakov Pacific Institute of Bioorganic Chemistry FEB RAS, Vladivostok, Russia; ^9^Department of Forestry and Natural Resources, National Ilan University, Ilan, Taiwan; ^10^Department of Medical Research, China Medical University Hospital, China Medical University, Taichung, Taiwan; ^11^Center for General Education, National Ilan University, Ilan, Taiwan

**Keywords:** NLRP3 inflammasome, *Litsea cubeba*, macrophages, dextran sulfate sodium-induced colitis, cytokines

## Abstract

The intracellular sensor NACHT, LRR, and PYD domain-containing protein 3 (NLRP3) inflammasome controls caspase-1 activity and the maturation and release of the cytokines interleukin (IL)−1β and IL−18. The NLRP3 inflammasome has attracted the attention of the pharmaceutical industry because it promotes the pathogenesis of many diseases, making it a promising target for drug development. *Litsea cubeba* (Lour.) is a plant traditionally used as a seasoning in Taiwan and in other Asian countries. In this study, we investigated the inhibitory activity of the leaves of *L. cubeba* against the NLRP3 inflammasome. We found that the ethanol extract of *L. cubeba* leaves (MLE) inhibited the NLRP3 inflammasome in macrophages by reducing caspase−1 activation and IL−1β secretion. MLE reduced pyroptosis in macrophages and inhibited the release of NLRP3 and apoptosis-associated speck-like protein containing a CARD (ASC). In a mechanistic study, MLE reduced mitochondrial reactive oxygen species (ROS) production and preserved mitochondrial integrity, which led to reduced mitochondrial DNA release into the cytosol. MLE did not reduce the expression levels of NLRP3, IL−1β precursor or TNF-α in lipopolysaccharide (LPS)-activated macrophages. These results indicated that MLE inhibited the NLRP3 inflammasome by suppressing the activation signals of the NLRP3 inflammasome but not by reducing the priming signal induced by LPS. In addition, oral administration of MLE (20−80 mg/kg) ameliorated dextran sulfate sodium (DSS)−induced colitis in a mouse model. Notably, mice that received MLE (1 and 2 g/kg) daily for 7 days did not exhibit visible side effects. Gas chromatography-mass spectrometry (GC-MS) analysis found that α-Terpinyl acetate (27.2%) and 1,8−Cineole (17.7%) were the major compounds in MLE. These results indicated that *L. cubeba* leaves have the potential to be a nutraceutical for preventing and improving NLRP3 inflammasome-related diseases.

## Introduction

The intracellular sensor NACHT, LRR, and PYD domain-containing protein 3 (NLRP3) inflammasome is a multiprotein complex composed of NLRP3, apoptosis-associated speck-like protein containing a caspase recruitment domain (ASC), and cysteine protease caspase−1 that controls caspase-1 activity and the maturation and release of the cytokines interleukin (IL)−1β and IL-18 (1). The NLRP3 inflammasome responds to medically relevant danger signals, including foreign molecules derived from pathogens and endogenous molecules associated with tissue damage or metabolic imbalances (2). Full activation of the NLRP3 inflammasome requires both a priming signal from pathogen-associated molecular patterns and an activation signal from a second stimulus, e.g., damage-associated molecular patterns, the former controlling the expression of NLRP3 and IL-1β precursor and the latter controlling caspase-1 activation (3). Aberrant activation of the NLRP3 inflammasome can be detected in many diseases. Pharmaceutical or genetic inhibition of NLRP3 prevents or improves these diseases. Aberrant activation of the NLRP3 inflammasome promotes the pathogenesis of inflammatory diseases, including chronic kidney disease, type II diabetes, atherosclerosis, neurodegenerative diseases, inflammatory bowel disease, gout, rheumatoid arthritis, cancers, and some infectious diseases (2).

The importance of the NLRP3 inflammasome has attracted the interest of researchers and biotech companies, and the field of NLRP3 inflammasome research is booming. Increasing evidence shows that the NLRP3 inflammasome is a promising therapeutic target in many diseases (4). Inhibition of the NLRP3 inflammasome did not dampen the broader immune responses needed to fight infection because the host defense ability can be covered by other inflammasomes, offering practical, effective, and safe therapy. The development of NLRP3 inhibitors has become an important topic in the pharmaceutical industry and scientific community (5). Although no NLRP3-targeting drugs have yet hit the market, various pharmaceutical companies are taking a wide range of strategies to tackle the NLRP3 inflammasome.

*Litsea cubeba* (Lour.) belongs to the family Lauraceae and genus Litsea. *L. cubeba* is a deciduous shrub with perennial rootstock, mostly distributed in the low-elevation mountain slopes of the central and eastern regions in Taiwan. The whole plant has a pungent spicy ginger scent. The indigenous Taiwanese call *L. cubeba* “Makauy.” Makauy is a traditional seasoning and medicinal plant used by indigenous people in Taiwan. The fruit is the most widely used part of *L. cubeba* and has a variety of biological activities. *L. cubeba* fruit essential oil inhibited the growth of drug-resistant *Staphylococcus aureus* and *Acinetobacter baumannii* (6, 7). *L. cubeba* fruit essential oil also inhibited the expression levels of tumor necrosis factor-α (TNF-α) and IL-12 in bacterial endotoxin (lipopolysaccharide, LPS)-stimulated bone marrow-derived dendritic cells and reduced contact hypersensitivity responses in mice (8). A new diterpene, cubelin, isolated from a methanol extract of *L. cubeba* fruits induced apoptosis in HeLa cells (9). *L. cubeba* fruit chloroform extracts showed insecticidal activities against Sitophilus zeamais Motschulsky (10). In our previous studies, we demonstrated that citral, a major compound in the *L. cubeba* fruit essential oil, improved focal segmental glomerulosclerosis (11) and lupus nephritis (12) in mice by reducing renal inflammation.

In recent years, the unique flavor of *L. cubeba* fruits has attracted public attention. However, insufficient production of *L. cubeba* fruits leads to price increases and restricts industrial development. Leaves are a sustainable and renewable resource; however, the leaves are currently the unused part of *L. cubeba*. In this study, we tested the inhibition potential of the ethanolic extract of *L. cubeba* leaves against the NLRP3 inflammasome in macrophages and in a mouse model of dextran sulfate sodium (DSS)-induced colitis.

## Materials and methods

### Reagents

*Escherichia coli* LPS and protease inhibitor cocktail were purchased from Sigma-Aldrich (St. Louis, MO). The NLRP3 activator ATP, nigericin and monosodium urate (MSU) were purchased from InvivoGen (San Diego, CA). Antibodies against IL-1β were purchased from R&D Systems (Minneapolis, MN). Antibodies against NLRP3 and caspase-1 were purchased from Adipogen Life Science (San Diego, CA). Antibodies against ASC, pro-IL-1β and actin were purchased from Santa Cruz Biotechnology (Santa Cruz, CA). ELISA kits, MitoSOX, MitoTracker Deep Red and MitoTracker Green were purchased from Thermo Fisher Scientific (Waltham, MA). The CytoScan LDH Cytotoxicity Assay kit was purchased from G-Bioscience (St. Louis, MO). DSS (MW: 36,000–50,000) was purchased from MP Biomedicals (LLC, France). RIPA lysis buffer, polyvinylidenedifloride membrane and RapidStep ECL Reagent were purchased from EMD Millipore (Darmstadt, Germany).

### Preparation of the ethanol extract of *Litsea cubeba* leaves

The *L. cubeba* leaves were collected from Lunpi, Datong Township, Yilan County 267002, Taiwan. The leaves were rinsed thoroughly and air-dried. Air-dried leaves (1,000 g) were extracted with 10 L of 95% methanol for 3 days at room temperature, which was repeated five times. The extract was filtered and concentrated to give an ethanolic extract of 200 g, named the Makauy leaf ethanol extract (MLE).

### MEL analysis by gas chromatography-mass spectrometry

The analytical gas chromatography (GC)-flame ionization detector (FID) and GC-mass spectrometry (MS) were used to analyze the major components of MLE as described previously with slight modifications (13). In the GC-FID analysis, briefly, a Hewlett-Packard 6,890 gas chromatograph with a DB-5 capillary columns (5% Phenyl/95% methylpolysiloxane, 30 m × 0.25 mm × 0.25 μm film thickness) was used. The MLE components were quantitatively determined by the FID. The oven heat program was 50°C for 2 min, then increase to 250°C at 5°C per min. The temperatures of injector and detector were heated to 270 and 250°C, respectively. The split ratio was 1:10 and hydrogen was used as carrier gas with a flow rate of 1 ml/min. One μl MLE sample [MLE/ethyl acetate 1:100 (v/v)] was injected in split mode. The linear retention indices of compounds were calculated with reference to a homologous series of C_8_-C_30_ *n*-alkanes (14). Relative proportions of compounds were calculated based on GC-FID peak areas measured on the DB-5 capillary column without use of correction factor. In the GC-MS analysis, briefly, a Hewlett-Packard 6,890/5,973 GG-MS system with a DB-5 capillary columns (the same parameters used in the GC-FID analysis) was used. Helium was used as carrier gas with a flow rate of 1 ml/min. The MS conditions were ionization voltage 70 eV, full scan mode: scan time: 0.3 s, and mass range was m/z 30–500. Compounds identification were based on calculated linear retention indices comparison with previous reports (14–16) or with the standard pure compounds and by comparison of their MS with those obtained in the NIST library “NIST 17” and “WILEY 11,” and in some components by co-injection with standard pure compounds.

### Cell culture

Mouse macrophage J774A.1 cells were purchased from the American Type Culture Collection (Rockville, MD). Cells were cultured in RPMI-1640 medium with 10% heat-inactivated fetal bovine serum at 37°C in a 5% CO_2_ incubator.

### Inhibition of the NLRP3 inflammasome by makauy leaf ethanol extract

Cells were primed with 1 μg/ml LPS for 4 h followed by incubation with 12.5, 25, or 50 μg/ml MLE or vehicle (0.1% DMSO) for 0.5 h. MLE was dissolved in DMSO to make stock solution and 0.1% volume of MLE stock was added to the cultures to make the working concentration. Cells were then incubated with 5 mM ATP for 0.5 h, 10 μM nigericin for 0.5 h, or 100 μg/ml MSU for 24 h. The levels of IL-1β in the supernatants were measured by ELISA as described previously with slight modifications (17). Briefly, prepared a mixture containing 50 μl of supernatants and 50 μl of biotinylated antibody. The mixture was added to a IL-1β antibodies pre-coated well and incubated for 2 h. After washes with washing buffer [phosphate-buffered saline (PBS) with 0.1% Tween 20], 100 μl of diluted HRP-conjugated streptavidin concentrate was added to each well and incubated for 0.5 h. After wash, 100 μl of a premixed tetramethylbenzidine substrate solution was added to each well and incubated for 0.5 h in the dark. The reaction was stopped by adding 100 μl of 2 M H_2_SO_4_ to each well, and the absorbance was read in an ELISA reader at 450 nm. The levels of pro-IL-1β/IL-1β and pro-caspase-1 (p45)/active caspase-1 (p10) in the supernatants were measured by western blotting as described previously with slight modifications (18). Briefly, protease inhibitor cocktail contained RIPA lysis buffer was used to lyse the cells. 30 μg protein of each samples were separated by SDS-polyacrylamide gel electrophoresis and then transferred to a polyvinylidenedifloride membrane. The membranes were blocked with blocking buffer (5% non-fat milk in PBS with 0.1% Tween 20) at room temperature for 2 h, and then were incubated with primary and HRP-conjugated secondary antibody in blocking buffer at room temperature for 1 and 0.5 h, respectively. After washes with washing buffer (PBS with 0.1% Tween 20), the membrane was developed using a RapidStep ECL Reagent. The signals were acquired using the GE Healthcare Life Sciences Amersham Imager 600 image system (Chicago, IL).

### Cytotoxic effect of makauy leaf ethanol extract

Cells were incubated with 12.5, 25, 50, 100, or 200 μg/ml MLE or 0.1% DMSO (vehicle) for 24 h. The cell numbers were calculated by the Trypan Blue exclusion test. For the LDH release assay, cells were incubated with 12.5, 25, 50, 100, or 200 μg/ml MLE, lysis buffer (maximum LDH release), or 0.1% DMSO (spontaneous LDH release) for 24 h. The LDH levels in the supernatants were measured by a CytoScan LDH Cytotoxicity Assay kit. The LDH release% was calculated as 100 X (sample OD − spontaneous OD)/(maximum OD − spontaneous OD).

### Inhibition of pyroptosis by makauy leaf ethanol extract

Cells were primed with 1 μg/ml LPS for 4 h followed by incubation with 12.5, 25, or 50 μg/ml MLE or vehicle (0.1% DMSO) for 0.5 h. Cells were then incubated with 5 mM ATP for 0.5 h. The levels of LDH in the supernatants were measured by the LDH release assay. The levels of NLRP3 and ASC in the supernatants were measured by western blotting.

### Inhibition of lipopolysaccharide-mediated responses by makauy leaf ethanol extract

Cells were incubated with 12.5, 25, or 50 μg/ml MLE or vehicle (0.1% DMSO) for 0.5 h, followed by incubation with 1 μg/ml LPS for 6 h. The levels of TNF-α and IL-6 in the supernatants were measured by ELISA. The levels of NLRP3 and proIL-1β in the cell lysates were measured by western blotting.

### Reduction of mitochondrial damage by makauy leaf ethanol extract

Cells were primed with 1 μg/ml LPS for 4 h, followed by incubation with 50 μg/ml MLE or vehicle (0.1% DMSO) for 0.5 h. Cells were then incubated with 5 mM ATP for 0.5 h. For the mitochondrial ROS production assay, cells were stained with 5 μM MitoSOX for 15 min. For the mitochondrial membrane integrity assay, cells were stained with 25 nM MitoTracker Deep Red and 25 nM MitoTracker Green for 15 min. The fluorescent signals of MitoSOX and MitoTracker were acquired by flow cytometry. For the mitochondrial DNA release assay, the levels of mitochondrial DNA in the cytosol were measured by detection of cytochrome c oxidase I DNA in the cytosol using quantitative PCR. The protocol and primer sequences are shown in our previous study (19).

### Mouse model of dextran sulfate sodium-induced colitis

Six-week-old male C57BL/6JNal mice were purchased from The National Laboratory Animal Center (Taipei, Taiwan). The mice were housed in a room controlled for temperature (23 ± 3°C) and relative humidity (40–60%). Mice were acclimated in the animal facility for a week before the experiments. Animal experiments were performed with the approval of the Institutional Animal Care and Use Committee of the National Ilan University (approval number: No. 102-40). The mice were randomized into 7 groups: Group I, vehicle + H_2_O, received normal drinking water with oral administration of vehicle daily, *n* = 5; Group II, vehicle + DSS, received 3% (wt/vol) DSS in drinking water with oral administration of vehicle daily, *n* = 5; Group III, DSS + 20 mg/kg MLE, received 3% DSS in drinking water with oral administration of 20 mg/kg MLE daily, *n* = 5; Group IV, DSS + 40 mg/kg MLE, received 3% DSS in drinking water with oral administration of 40 mg/kg MLE daily, *n* = 5; Group V, DSS + 80 mg/kg MLE, received 3% DSS in drinking water with oral administration of 80 mg/kg MLE daily, *n* = 5; Group VI, 80 mg/kg 5-ASA + DSS, received 3% DSS in drinking water with oral administration of 80 mg/kg 5-ASA daily, *n* = 5; and Group VII, 80 mg/kg MLE + H_2_O, received normal drinking water with oral administration of 80 mg/kg MLE daily, *n* = 5. For DSS treatment, mice were treated with 3% DSS in drinking water *ad libitum* for 7 days, followed by normal water for 1 day. MLE or 5-ASA was administered intragastrically daily during DSS treatment. The body weight of each mouse was recorded daily. On Day 8 following induction with DSS, mice were sacrificed for the collection of colonic tissue and spleen.

### Analysis of the levels of IL-1β and IL-6 in colons

The total colon protein from mice in each group was extracted by homogenization with lysis buffer and centrifuged at 13,000 rpm at 4°C for 20 min. The supernatants were taken, and the protein concentration was determined by the BCA protein assay before IL-1β and IL-6 ELISA analysis.

### Safety evaluation of mice exposed to makauy leaf ethanol extract

Six-week-old male C57BL/6JNal mice were intragastrically administered 1 or 2 g/kg MLE or vehicle daily for 7 days. The mice were sacrificed for organ collection on Day 21 post-administration. The body weight of each mouse was recorded daily before sacrifice.

### H&E analysis

The histological analysis was performed as described previously with slight modifications (20). Part of the colon was fixed in 10% buffered formalin and embedded in paraffin. Sections were stained with H&E according to standard protocols conducted by Energenesis Biomedical Co., Ltd. (Taipei, Taiwan).

### Statistical analysis

Two-tailed *t*-tests and ANOVA with Dunnett’s multiple comparisons test were used for statistical analysis for two groups and three or more groups, respectively. Error bars represent the standard deviation of three separate experiments. *, ^**^, and ^***^ represent *p* < 0.05, *p* < 0.01, and *p* < 0.001, respectively.

## Results

### Makauy leaf ethanol extract inhibits the NLRP3 inflammasome

**FIGURE 1:**
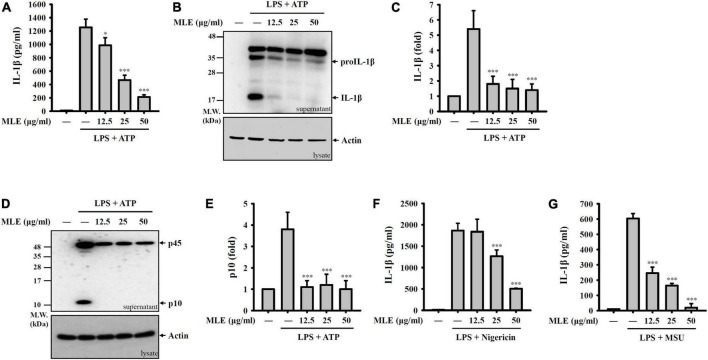
MLE inhibits the NLRP3 inflammasome. **(A–E)** LPS-primed J774A.1 macrophages were incubated with MEL for 0.5 h before ATP stimulation for an additional 0.5 h. The levels of IL-1β in the supernatants were analyzed by ELISA **(A)** and western blot **(B)**. **(C)** The column diagram represents the fold change of IL-1β in **(B)** compared with the control group analyzed by ImageJ. **(D)** The levels of pro-caspase-1 (p45) and active caspase-1 (p10) in the supernatants were analyzed by western blot. **(E)** The column diagram represents the fold change of p10 in **(D)** compared with the control group analyzed by ImageJ. **(F,G)** LPS-primed J774A.1 macrophages were incubated with MEL for 0.5 h before nigericin stimulation for an additional 0.5 h **(F)** or MSU stimulation for an additional 24 h **(G)**. The levels of IL-1β in the supernatants were analyzed by ELISA. The western blot images are representative of three different experiments. The data are expressed as the mean ± SD of three separate experiments. *p < 0.05 and ***p < 0.001 compared to NLRP3 inflammasome-activated cells.

To investigate whether MLE inhibits the activation of the NLRP3 inflammasome, macrophages were primed with LPS for 4 h followed by incubation with MLE for 0.5 h. Mouse J774A.1 macrophages were then stimulated with ATP for an additional 0.5 h. The concentration of IL-1β in the culture medium was analyzed by ELISA. We found that MLE reduced ATP-mediated IL-1β expression in a dose-dependent manner (Figure 1A). The IL-1β inhibitory activity of MLE was confirmed by the detection of IL-1β in the culture medium using western blotting (Figures 1B,C). MLE also inhibited active caspase-1 (p10) expression in the culture medium, as analyzed by western blotting, indicating that MLE inhibited caspase-1 activation (Figures 1D,E). In addition, MLE not only reduced ATP-mediated IL-1β expression but also reduced IL-1β expression induced by other NLRP3 activators, including nigericin (Figure 1F) and MSU (Figure 1G). These results indicate that MLE inhibits the NLRP3 inflammasome in macrophages.

### Makauy leaf ethanol extract inhibits pyroptosis

**FIGURE 2:**
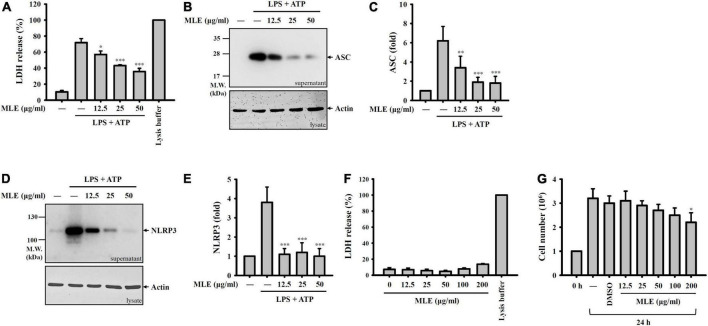
MLE inhibits pyroptosis. **(A–E)** LPS-primed J774A.1 macrophages were incubated with MEL for 0.5 h before ATP stimulation for an additional 0.5 h. The levels of LDH in the supernatants were analyzed by an LDH release kit **(A)**, and the levels of ASC in the supernatants were analyzed by western blot **(B)**. **(C)** The column diagram represents the fold change of ASC in **(B)** compared with the control group analyzed by ImageJ. **(D)** The levels of NLRP3 in the supernatants were analyzed by western blot. **(E)** The column diagram represents the fold change of NLRP3 in **(D)** compared with the control group analyzed by ImageJ. **(F)** J774A.1 macrophages were incubated with MEL for 24 h, and the levels of LDH in the supernatants were analyzed by an LDH release kit. **(G)** J774A.1 macrophages were incubated with MEL for 24 h, and the cell numbers were calculated by the Trypan Blue exclusion test of cell viability. The western blot images are representative of three different experiments. The LDH and cell counting data are expressed as the mean ± SD of three separate experiments. *p < 0.05, **p < 0.01, and ***p < 0.001 compared to the LPS + ATP group in **(A–E)** or compared to the vehicle-treated group in **(G)**.

Pyroptosis is an inflammatory form of cell death caused by caspase-1-dependent plasma membrane pore formation, which leads to an increase in plasma membrane permeability and the release of inflammatory intracellular contents (21–23). We found that LDH was released from ATP-stimulated macrophages, and this effect was reduced by MLE (Figure 2A). NLRP3 inflammasome components released from pyroptotic macrophages serve as particulate danger signals that amplify the inflammatory response (24). We found that ATP induced ASC (Figures 2B,C) and NLRP3 (Figures 2D,E) release, and these effects were reduced by MLE. Importantly, MLE treatment for 24 h did not cause cytotoxicity to macrophages, as demonstrated by the LDH release assay (Figure 2F). Although MLE did not cause cytotoxicity to macrophages, at high concentrations, it slightly reduced cell growth (Figure 2G).

### Makauy leaf ethanol extract did not inhibit the priming signals of the NLRP3 inflammasome

**FIGURE 3:**
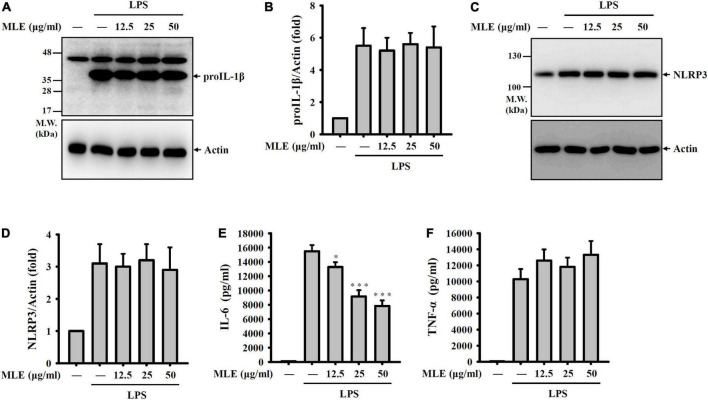
MLE did not inhibit the priming signals of the NLRP3 inflammasome. **(A–F)** J774A.1 macrophages were incubated with MEL for 0.5 h before LPS stimulation for an additional 6 h. **(A)** The levels of proIL-1β in the cell lysates were analyzed by western blot. **(B)** The column diagram represents the fold change of proIL-1β in **(A)** compared with the control group normalized to actin analyzed by ImageJ. **(C)** The levels of NLRP3 in the cell lysates were analyzed by western blot. **(D)** The column diagram represents the fold change of NLRP3 in **(C)** compared with the control group normalized to actin analyzed by ImageJ. The levels of IL-6 **(E)** and TNF-α **(F)** in the supernatants were analyzed by ELISA. The western blot images are representative of three different experiments. The ELISA data are expressed as the mean ± SD of three separate experiments. *p < 0.05 and ***p < 0.001 compared to the LPS group.

To investigate how MLE inhibits the NLRP3 inflammasome, the effect of MLE on the priming signals of the NLRP3 inflammasome was tested. Macrophages were incubated with MLE for 0.5 h followed by LPS stimulation for 6 h. The expression levels of proIL-1β and NLRP3 in the cell lysates were analyzed by western blot. We found that LPS increased the expression levels of proIL-1β (Figures 3A,B) and NLRP3 (Figures 3C,D); however, MLE did not affect proIL-1β and NLRP3 expression. In addition, MLE reduced the expression of IL-6 (Figure 3E) but not TNF-α (Figure 3F) in LPS-activated macrophages. These results indicate that MLE inhibited the NLRP3 inflammasome not through reducing the priming signals induced by LPS.

### Makauy leaf ethanol extract inhibited NLRP3 inflammasome activation signals by reducing mitochondrial damage

**FIGURE 4:**
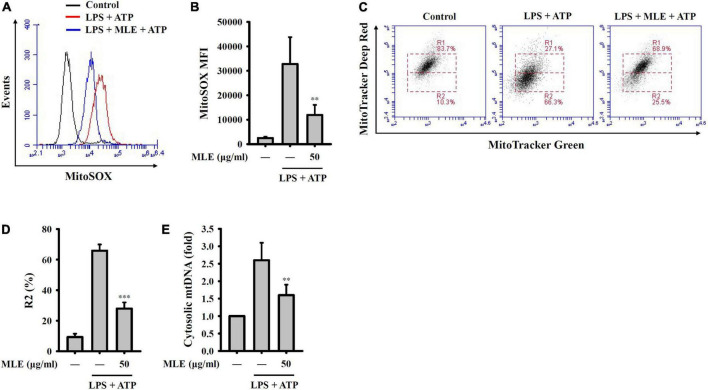
MLE inhibited activation signals of the NLRP3 inflammasome by reducing mitochondrial damage. **(A–E)** LPS-primed J774A.1 macrophages were incubated with MEL for 0.5 h before ATP stimulation for an additional 0.5 h. **(A)** Mitochondrial ROS production was analyzed by MitoSOX staining using flow cytometry. **(B)** The column diagram represents the mean fluorescence intensity of MitoSOX in **(A)**. **(C)** Mitochondrial membrane integrity was analyzed by MitoTracker Deep Red and Green staining using flow cytometry. **(D)** The column diagram represents the% of low MitoTracker Deep Red signal (R2) in **(C)**. **(E)** Mitochondrial DNA release into the cytosol was analyzed by the detection of cytochrome c oxidase I DNA in the cytosol. The data are expressed as the mean ± SD of three separate experiments. **p < 0.01 and ***p < 0.001 compared to the LPS + ATP group.

NLRP3 activator stimulation leads to the release of reactive oxygen species (ROS) from mitochondria and is an important step for downstream caspase-1 activation. We found that MLE reduced mitochondrial ROS generation in ATP-stimulated macrophages, as analyzed by staining for the mitochondrial ROS indicator MitoSOX (Figures 4A,B). Mitochondrial ROS cause mitochondrial membrane damage, which induces mitochondrial DNA release into the cytosol. The released mitochondrial DNA binds to NLRP3 and activates caspase-1 (25). We demonstrated that MLE reduced mitochondrial membrane integrity loss in ATP-stimulated macrophages, as analyzed by the mitochondrial membrane potential indicator MitoTracker Deep Red and Green staining (Figures 4C,D). In addition, we found that the translocation of mtDNA into the cytosol was reduced by MLE (Figure 4E). These results indicated that MLE inhibits the NLRP3 inflammasome partially by preventing mitochondrial damage.

### Makauy leaf ethanol extract ameliorates dextran sodium sulfate-induced colitis in a mouse model

**FIGURE 5:**
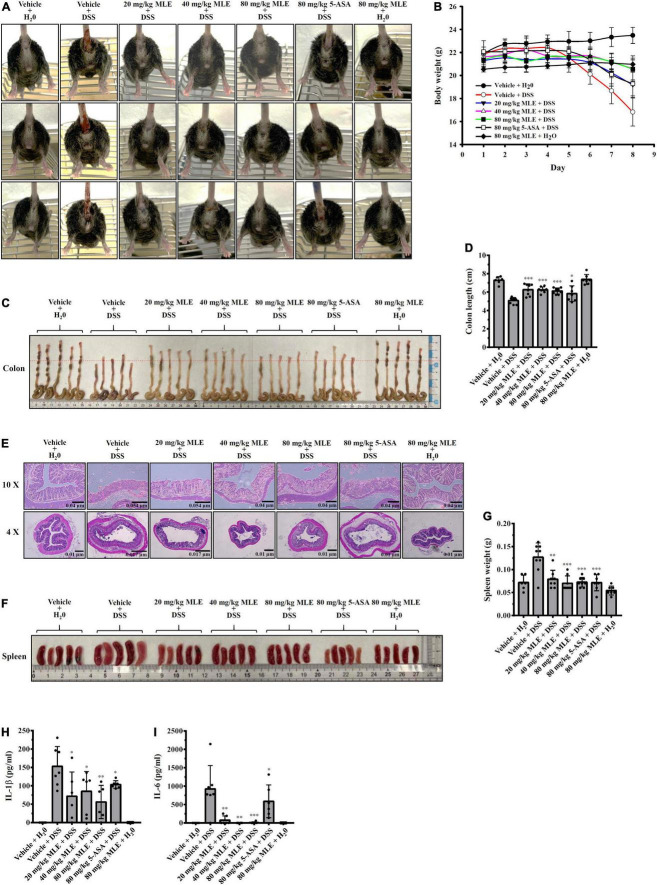
MLE ameliorates DSS-induced colitis in a mouse model. **(A)** Images of representative diarrhea and bloody stool from mice. **(B)** Effect of MLE on body weight loss. **(C)** Effect of MLE on colonic shortening. **(D)** The column diagram represents the colon length in **(C)**. **(E)** Effect of MLE on colonic damage analyzed by H&E staining. **(F)** Effect of MLE on splenomegaly. **(G)** The column diagram represents the spleen weight in **(F)**. **(H)** Effect of MLE on the levels of IL-1β in colon tissue. **(I)** Effect of MLE on the levels of IL-6 in colon tissue. The data are expressed as the mean ± SD. *, **, and *** indicate a significant difference at the level of *p* < 0.05, *p* < 0.01, and *p* < 0.001, respectively, compared to vehicle + DSS mice.

It has been demonstrated that the NLRP3 inflammasome plays a crucial role in the pathogenesis of IBD (26). To investigate the *in vivo* inflammatory bowel disease inhibition potential of MLE, we tested the effect of MLE on a mouse model of DSS-induced colitis, characterized by significant NLRP3 inflammasome activation in colon tissue. We found that DSS caused significant diarrhea and bloody stool, and these effects were significantly improved by oral administration of 20–80 mg/kg MLE or 80 mg/kg 5-aminosalicylic acid (5-ASA), a clinical drug (Figure 5A). As DSS caused diarrhea, it induced significant body weight loss in mice, and oral administration of MLE or 5-ASA attenuated the loss of body weight (Figure 5B). DSS also caused significant colonic shortening in mice, and MLE or 5-ASA significantly attenuated colonic shortening (Figures 5C,D). MLE improved the colonic damage analyzed by H&E staining (Figure 5E). In addition, DSS induced significant splenomegaly, indicating the elevated inflammatory status in mice, and MLE or 5-ASA significantly ameliorated the splenomegaly induced by DSS (Figures 5F,G). Furthermore, we found that DSS significantly increased the levels of IL-1β (Figure 5H) and IL-6 (Figure 5I) in colon tissue, and these effects were significantly reduced by MLE or 5-ASA, indicating that MLE ameliorates DSS-induced colonic inflammation.

### Preliminary safety evaluation of mice exposed to makauy leaf ethanol extract

**FIGURE 6:**
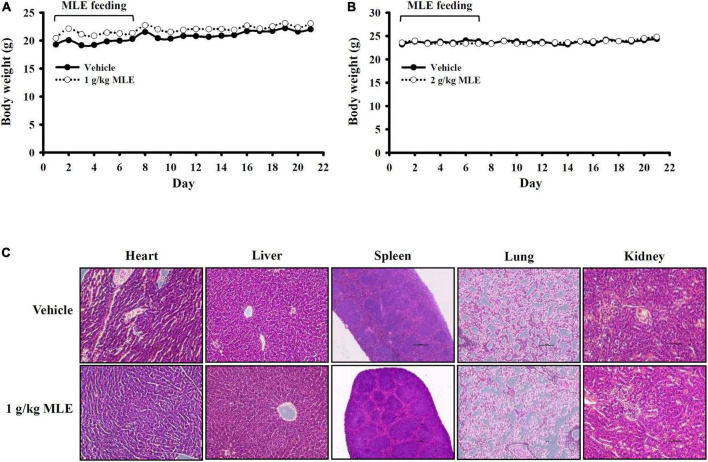
Preliminary safety evaluation of mice exposed to MLE. **(A,B)** Effect of MLE on body weight. **(C)** H&E staining of tissue sections from vehicle- or MLE-fed mice.

Oral administration of 1 or 2 g/kg MLE daily for 7 days did not cause body weight loss in mice, and no side effects were observed until 21 days after the first administration (Figures 6A,B). The organs were collected from the MLE-fed mice at 21 days after the first administration, and no pathological changes in the heart, liver, spleen, lung, or kidney were observed (Figure 6C). These results indicate that MLE was found to be safe for mice under this condition.

### Identification of compounds from makauy leaf ethanol extract using gas chromatography-mass spectrometry Analysis

**FIGURE 7:**
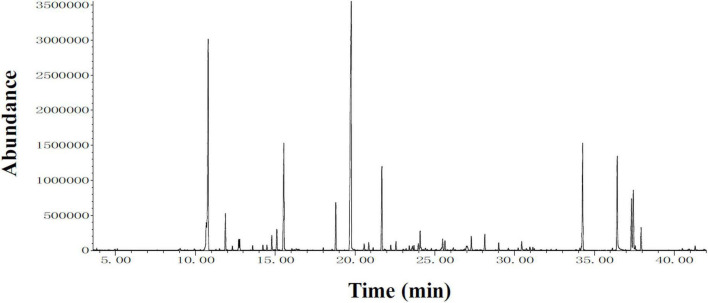
Chromatograms obtained from GC-MS screening of MLE.

To identify the compounds that can be used for quality control, GC-MS analysis of MLE was performed (Figure 7), and the identified compounds in the MLE were summarized in Table 1. The top 10 major compounds of MLE were α-Terpinyl acetate (27.2%), 1,8-Cineole (17.7%), α-Terpineol (7.4%), Ethyl hexadecanoate (6.4%), Phytol (6.1%), Limonene (5.6%), β-Caryophyllene (4.9%), Ethyl 9,12,15-octadecatrienoate (4.2%), Linoleic acid ethyl ester (3.0%), and Terpinen-4-ol acetate (2.5%).

**TABLE 1:** Chemical compositions of MLE analyzed by GC-MS.

Compound I.D.	LRI_Exp_^a^	LRI_Lit_^b^	Concentration (%)	Identification^c^
Sabinene	974	975	0.2	MS, LRI, CO-ST
*p*-Mentha-1(7),8-diene	1,002	1,004	0.1	MS, LRI
Limonene	1,029	1,029	5.6	MS, LRI, CO-ST
1,8-Cineole	1,033	1,031	17.7	MS, LRI, CO-ST
γ-Terpinene	1,057	1,059	0.1	MS, LRI, CO-ST
*cis*-Sabinene hydrate	1,069	1,070	1.8	MS, LRI
Terpinolene	1,086	1,088	0.2	MS, LRI, CO-ST
Linalool	1,094	1,096	0.6	MS, LRI, CO-ST
*trans*-Sabinene hydrate	1,096	1,098	0.6	MS, LRI
*trans*-*p*-Mentha-2,8-dien-1-ol	1,125	1,122	0.2	MS, LRI
Citronellal	1,150	1,153	0.3	MS, LRI, CO-ST
δ-Terpineol	1,166	1,166	0.9	MS, LRI
Terpinen-4-ol	1,176	1,177	1.2	MS, LRI, CO-ST
α-Terpineol	1,190	1,189	7.4	MS, LRI, CO-ST
Bornyl acetate	1,287	1,288	0.1	MS, LRI, CO-ST
Terpinen-4-ol acetate	1,301	1,299	2.5	MS, LRI
α-Terpinyl acetate	1,346	1,349	27.2	MS, LRI, CO-ST
β-Elemene	1,388	1,390	0.4	MS, LRI, CO-ST
β-Caryophyllene	1,416	1,419	4.9	MS, LRI, CO-ST
(*e*)-Isoeugenol	1,450	1,451	0.3	MS, LRI
α-Humulene	1,451	1,454	0.5	MS, LRI, CO-ST
*cis*-Muurola-4(14),5-diene	1,469	1,466	0.1	MS, LRI
γ-Muurolene	1,476	1,479	0.1	MS, LRI
β-Selinene	1,489	1,490	0.3	MS, LRI
α-Muurolene	1,499	1,500	0.4	MS, LRI, CO-ST
(e,e)-α-Farnesene	1,502	1,505	0.3	MS, LRI
γ-Cadinene	1,510	1,513	0.4	MS, LRI
δ-Cadinene	1,521	1,523	1.2	MS, LRI
Caryophyllene oxide	1,580	1,583	0.6	MS, LRI, CO-ST
t-Cadinol	1,638	1,640	0.2	MS, LRI, CO-ST
t-Muurolol	1,641	1,642	0.6	MS, LRI, CO-ST
α-Cadinol	1,652	1,654	0.8	MS, LRI, CO-ST
Ethyl hexadecanoate	1,990	1,993	6.4	MS, LRI
Ethyl heptadecanoate	2,086	2,089	0.1	MS, LRI
Phytol	2,097	2,110	6.1	MS, LRI, CO-ST
Linoleic acid ethyl ester	2,161	2,162	3.0	MS, LRI
Ethyl 9,12,15-octadecatrienoate	2,170	2,169	4.2	MS, LRI
Ethyl Oleate	2,173	2,174	0.3	MS, LRI
Octadecanoic acid, ethyl ester	2,192	2,193	1.3	MS, LRI
Eicosanoic acid, ethyl ester	2,392	2,395	0.3	MS, LRI

^a^LRI_Exp_ = Determined LRI relative to n-alkanes (C_8_-C_30_) on DB-5 capillary column. ^b^LRI_Lit_ = LRI on DB-5 capillary column from reference 14. ^c^Identification by MS = NIST 17 and Wiley 11 libraries spectra, and the literatures; LRI was same as references 14–16. CO-ST, co-injection and comparison with the l LRI and mass spectra of standards.

## Discussion

Traditionally, the bacterial endotoxin LPS has been used as the stimulus to induce an inflammatory response in an inflammatory model system. Although this model is a commonly used anti-inflammatory drug screening platform, the disadvantage is that the inflammatory response induced by LPS cannot represent a specific disease condition. The NLRP3 inflammasome has become an important research topic, as it can be activated by particular medically relevant stimuli and is a promising target for drug discovery (4, 5). MCC950, a potent and selective small-molecule inhibitor of NLRP3, inhibited canonical and non-canonical NLRP3 activation and improved various NLRP3-associated diseases in animal models of inflammatory bowel disease, neurodegenerative diseases, cardiovascular diseases, rheumatoid arthritis, lung inflammation, asthma, stroke, liver disease, and many other inflammatory conditions (27, 28). However, the human trials of MCC950 were stopped as the blood levels of a liver enzyme were raised. Although no NLRP3 inflammasome inhibitor has been approved for marketing, the development of NLRP3 inflammasome inhibitors with few adverse drug reactions has attracted the interest of the pharmaceutical industry and academic researchers (4, 5).

Natural products are important sources for drug development because they have a wide range of diverse multidimensional chemical structures (29, 30). We identified several natural products that inhibited the NLRP3 inflammasome *in vitro* and ameliorated NLRP3-associated disorders *in vivo*. We demonstrated that citral (3,7-dimethyl-2,6-octadienal) isolated from *L. cubeba* inhibited the production of nitric oxide (NO), TNF-α, and IL-6 by reducing NF-κB activation and ROS production in LPS-activated RAW264.7 macrophages and improved focal segmental glomerulosclerosis in mice (11). We further demonstrated that citral inhibited ATP-induced caspase-1 activation and IL-1β production in LPS-primed J774A.1 macrophages and alleviated lupus nephritis in mice (12). Autophagy is an important self-protective mechanism that can inhibit activation of the NLRP3 inflammasome by preserving mitochondrial integrity (31). We found that honokiol (32) from *Magnolia officinalis* and resveratrol in the skin of red grapes (19) inhibited the NLRP3 inflammasome in macrophages and alleviated renal inflammation in mice through autophagy induction. In this study, we demonstrated that MLE reduced mitochondrial ROS production and mitochondrial membrane integrity loss and inhibited the NLRP3 inflammasome in macrophages; however, the effect of MLE on autophagy needs further investigation.

Although most of the studies focus on the fruits of *L. cubeba*, the roots, bark, wood, twigs, and leaves of *L. cubeba* also exert biological functions. *L. cubeba* root ethanol extract improved adjuvant arthritis in rats by reducing the levels of proinflammatory mediators and increasing the anti-inflammatory cytokine IL-10 in serum (33). The same group further identified 9,9′-O-di-(E)-fer-uloyl-meso-5,5′-dimethoxysecoisolariciresin, a pure compound isolated from *L. cubeba* root ethanol extract, that inhibited LPS-induced NO and TNF-α expression in macrophages (34) and reduced RANKL-induced osteoclast differentiation in mouse bone marrow macrophages (35). Boldine and rozuline isolated from *L. cubeba* root ethanol extract inhibited xylene-induced ear edema in mice and carrageenan-induced paw edema in rats (36). Furthermore, *L. cubeba* bark methanol extract inhibited NO and PGE_2_ production in LPS-activated macrophages (37). Litebamine, a phenanthrene alkaloid from the wood of *L. cubeba*, exerts potential cardiovascular protective activity, as it inhibits rat smooth muscle cell adhesion and migration on collagen (38). A terpenoid ester glycoside isolated from *L. cubeba* twig ethanol extract induced cell death in human non-small-cell lung carcinoma A549 cells and human ileocecal adenocarcinoma HCT-8 cells (39). 1,8-Cineole- or linalool-containing *L. cubeba* leaf essential oil exerted antibacterial activity against *Escherichia coli* (40). In this study, we are the first to show that the ethanolic extract of *L. cubeba* leaves exerts NLRP3 inflammasome inhibitory activity in macrophages and ameliorates DSS-induced colitis in mice.

Although *L. cubeba* fruit and its essential oil have been used as seasonings for a long time, toxicity should be considered. The studies performed in Institute of Cancer Research mice and Sprague-Dawley rats showed that *L. cubeba* fruit essential oil is slightly toxic, as the oral 50% lethal dose is approximately 4,000 mg/kg of body weight. Importantly, no genetic toxicity was observed using standard genetic toxicity testing (41). *L. cubeba* leaves have also been used as seasonings or tea for many years; however, there is no report evaluating the toxicity of *L. cubeba* leaves. Here, we provide preliminary results showing that there were no side effects observed in mice orally administered *L. cubeba* leaf ethanol extract at 1 or 2 g/kg body weight once daily for 7 continuous days. However, the detailed toxicity test, including the genetic toxicity of *L. cubeba* leaves, should be investigated before application in the food industry. It has been reported that α-terpinyl acetate, the major compound of MLE, reduced TNF-α and IL-6 in LPS-activated macrophages (42). In addition, 1,8-cineole, another major compound of MLE inhibited the NLRP3 inflammasome activation in macrophages (43). The limitation of this study is that although some compounds were identified, no active fractions or compounds with NLRP3 inflammasome inhibitory activity were identified. Activity-guided fractionation of MLE should be performed to isolate the active components in *L. cubeba* leaves in the future.

## Data availability statement

The original contributions presented in this study are included in the article/supplementary material, further inquiries can be directed to the corresponding author.

## Ethics statement

The animal study was reviewed and approved by the Institutional Animal Care and Use Committee of the National Ilan University.

## Author contributions

C-FW was the guarantor of the article. W-TW and C-FW conceived and designed the study. W-TW, C-HW, L-HL, D-YH, and H-WC performed the experiments and analyzed the data. H-TH, C-LH, and OC assisted with some experiments. S-MC, S-PY, and K-FH contributed to critical revision of the manuscript. W-TW, C-HW, and C-FW wrote and finished the manuscript. All authors participated in revising the manuscript and approved the final version.
